# Physiotherapeutic assistance *verse* home care assistance in the early rehabilitation of total knee arthroplasty during COVID-19 lockdown

**DOI:** 10.1186/s42836-020-00067-z

**Published:** 2021-03-02

**Authors:** Lavindra Tomar, Gaurav Govil, Pawan Dhawan

**Affiliations:** 1 Department of Orthopaedics, Max Super Speciality Hospital, Patparganj, Delhi, A-702 Vardhman apartment, Mayur Vihar Phase1extension, Delhi, 110091 India; 2 Department of Orthopaedics, Max Super Speciality Hospital, Patparganj, Delhi, D101, Sunshine Helios, Sector 78, Noida, Uttar Pradesh 201305 India; 3 Department of Orthopaedics, Max Super Speciality Hospital, Patparganj, Delhi, House no 37, Sukh Vihar, Delhi, 110051 India

**Keywords:** Total knee arthroplasty, Rehabilitation, Physiotherapy, Pandemic, Covid-19, Functional outcome, Patient-reported outcomes, Home care-giver, ***Level of Evidence:*** Ia.

## Abstract

**Background:**

The aim of this retrospective comparison study was to assess early functional recovery of total knee arthroplasty with home care assistance during COVID-19 lockdown.

**Methods:**

A total of 16 patients (27 knees involved) were divided into a pre-lockdown group (10 patients; 17 knees) and a post-lockdown group (6 patients, 10 knees) in terms of the time of surgeries performed before and after lockdown, respectively, due to COVID-19 pandemic. Patients of pre-lockdown group underwent rehabilitation under the guidance of trained physiotherapists for at-home sessions and under assisted physiotherapy. Patients of post-lockdown group followed the rehabilitation protocol of at-home sessions and under home-care assistance during COVID-19 lockdown. Functional recovery of the knee was assessed against the Knee Injury and Osteoarthritis Outcome Score, Junior. A *p* < 0.05 was considered statistically significant.

**Results:**

The pre- and postoperative mean KOOS Junior of pre-lockdown group were 48.73 ± 2.64 and 64.91 ± 2.74, respectively (*p* < 0.001*).* The pre- and postoperative scores of post-lockdown group were 48.83 ± 2.83 and 67.84 ± 4.31 (*p* < 0.001), respectively. Intergroup comparison between pre- and postoperative KOOS Jr. revealed no significant differences (*p* > 0.05).

**Conclusion:**

Although the COVID-19 lockdown affected the routine postoperative rehabilitation after total knee arthroplasty, the coordination among the surgeon, therapists, and home caregivers can provide sustained assistance in rehabilitation. The guidelines for practitioners and physiotherapists can benefit functional recovery of the knee.

## Introduction

Total knee arthroplasty (TKA) is a highly successful treatment for osteoarthritis [1, 2]. The success relies not only on the surgical techniques, but also on the postoperative therapies and rehabilitation [1]. The lockdown due to COVID-19 pandemic is a unique situation where no guidelines were available for patients’ postoperative management [2].

The COVID-19 pandemic has devastating social and health consequences. The governments around the world are all in the crisis-management mode. The Prime Minister of India announced a nationwide lockdown effective as of 25th March 2020 to prevent community transmission [3, 4]. This unmatched event of this magnitude affected both the surgeons and patients who underwent TKA. As the patients became ready for discharge from hospital, they faced additional obstacles in their path to recovery [5]. In fact, the lockdown affected the patients and trained personnel in the routine postoperative rehabilitation after TKA. Currently, no guidelines are available for practitioners and physiotherapists to follow and such unavailability further compounds the management dilemma.

We conducted a retrospective comparison study to assess the early functional recovery of TKA patients without assisted physiotherapy sessions after lockdown for at least 2 weeks. We also evaluated the efficacy of home-based care provision that ensured continued assistance in the rehabilitation during COVID-19 lockdown.

## Materials and methods

The institutional review boards of the participating hospitals reviewed the study and approved the protocol. Informed consent was obtained from each patient.

### Patient selection and allocation

A total of 29 TKAs were performed in 17 patients from January 2020 to March 2020. Our eligibility criteria were (1) patients with a confirmed diagnosis of knee arthritis; (2) involvement of single or both knees; (3) primary TKA; (4) Persisting pain and restricted activities of daily living; (5) failure to respond to conservative managements; and (6) ambulatory patients with or without a walking aid. Patients were excluded if any of the following conditions were present: (1) an index arthroplasty of opposite limb 3 months prior to the planned TKA; (2) surgeries of lower limbs affecting their gait pattern; and (3) presence of neuromuscular or neurodegenerative diseases. The patients were divided into a pre-lockdown (PRLD) group and a post-lockdown (POLD) group according to the surgeries performed before lockdown from January 2020 to 15th March 2020 and one-week preceding lockdown from 16th March 2020 to 25th March 2020, respectively, introduced due to COVID-19 pandemic.

### Surgery and post-operative management

In both groups, preoperative analgesic regimen included oral administration of acetaminophen, ibuprofen, and gabapentin. Operations were performed by the same senior surgeon and orthopaedic team and *as per* the similar standard operating protocol. After surgery, the patients were orally administered acetaminophen, ibuprofen, and gabapentin, and supplemental buprenorphine skin patches were used whenever needed. The patients received assisted physiotherapy sessions twice daily, which included ambulation with support and quadriceps strengthening exercises. The patients also received toilet and chair sitting training until discharge. The discharge instructions were patient-specific and were printed on leaflets, covering post-TKA rehabilitation exercises and dos and don’ts.

Patients in PRLD group underwent rehabilitation under the instruction of trained physiotherapists for at-home sessions. The guided and assisted physiotherapy was performed in the following 2 weeks thereafter.

Patients in POLD group followed the rehabilitation protocol of at-home sessions. Because the physiotherapy under a trained physiotherapist was impossible during lockdown, we first selected a home caregiver who assisted in physiotherapy for the next 2 weeks after lockdown was imposed. A person was selected as a home caregiver only if he or she had all of the following features: (a) spouse or other family member of the patient; (b) unpaid; (c) stayed with the patient in the hospital and home; (d) gave physical and emotional support for knee disability; and (e) participated in the management during lockdown period [6]. Other people were excluded as home caregivers, and their qualifications were not considered for inclusion. During patient hospitalization, the home caregiver was guided by a surgeon and trained by a physiotherapist according to the rehabilitation protocols. The home caregiver was given oral/visual instructions and taught on simulation methods two times a day, until the patient was discharged. We maintained a communication link to provide added assistance and guidance to the home caregiver, but there were no defined monitoring tools.

### Outcome evaluation

Preoperatively, we used the American Society of Anaesthesiologists grading to assess the physical status [7]. The body mass index was also assessed. After surgery, we evaluated the patients in PRLD group with regard to pain along the operated limb, wound healing, and swelling of limb at the time of stitch removal (15 days after index arthroplasty). We also assessed walking ability, capability of raising from a chair, and dependent activities of daily living. Six weeks after arthroplasty, the patients in PRLD group was assessed for early functional recovery. In order to minimize hospital visits in view of evolving pandemic, assessments were done either at hospital visit or by telecommunication *as per* the government guidelines. Functional recovery of the knee was assessed with the Knee Injury and Osteoarthritis Outcome Score, Junior (KOOS Jr). The KOOS Jr. included 7 questionnaires on joint-specific pain and physical function [8, 9]. It allowed for faster completion and greater patient engagement without any physical contact [10]. Complications, including infection, symptomatic deep vein thrombosis, dislocation, and mortality, were also assessed.

In POLD group, patients were apprised regarding the COVID-19 hospital policy. After surgery, the patient and the home caregiver were taught a standardized home-exercise program by experienced physiotherapists. This program consisted of simple exercises (strengthen lower-limb quadriceps, hamstrings, hip abductors and extensors) to improve the knee mobility, and advice regarding knee positioning and gait training [2]. The learning program was completed before discharge. In order to minimize hospital visits, similar assessments were performed through the telephone or video conversation [11]. The home caregiver and the same physiotherapist assessed the outcomes.

### Statistical analysis

Statistical analysis was done and the variables were compared using independent *t*-test and one-way ANOVA. A *p* < 0.05 was considered statistically significant. The data were inputted into Microsoft Excel spreadsheet and statistical analysis was done by using SPSS version 24 (SPSS, Inc., Chicago, Ill.).

## Results

In this study, 11 patients received bilateral TKAs and 6 patients underwent unilateral TKAs. Finally, 10 patients (17 TKAs) in PRLD group were analysed, because one patient (bilateral TKAs) was lost to the follow-up. Six patients (10 TKAs) in POLD group were analyzed. The clinical and demographic characteristics of both groups are detailed in Table 1. The age of PRLD and POLD groups ranged from 61 to 67 years and 56 to 63 years, respectively. The male to female ratios of two groups were similar. Fifteen patients were diagnosed with primary knee osteoarthritis, and one (unilateral TKA in PRLD group) was diagnosed as having rheumatoid arthritis.

**Table 1:** Demographic data and patient distribution of the two groups

Patient Parameters (Variables)		PRLD group	POLD group	***p*** Value
Age range (in years)		61–67	56–63	0.358
Sex	Male	3	2	–
	Female	7	4	
Side	Bilateral	7	4	–
	Unilateral	3	2	
TKA Indication	Primary OA	9	6	–
	Others	1 (RA)	0	
ASA Grade	1	0	1	
	2	3	1	
	3	7	4	
BMI		26.80 ± 2.67	28.46 ± 4.35	0.442

*OA* osteoarthritis, *RA* rheumatoid arthritis, *TKA* total knee arthroplasty, *ASA* American Society of Anaesthesiologists, *BMI* body mass index

**Fig. 1:**
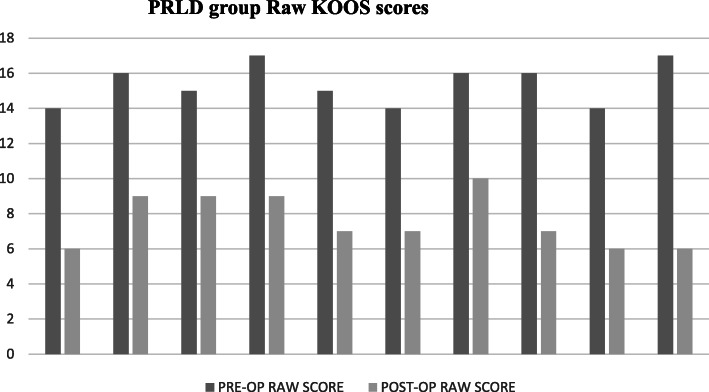
Pre- and postoperative KOOS Jr. of PRLD group

**Fig. 2:**
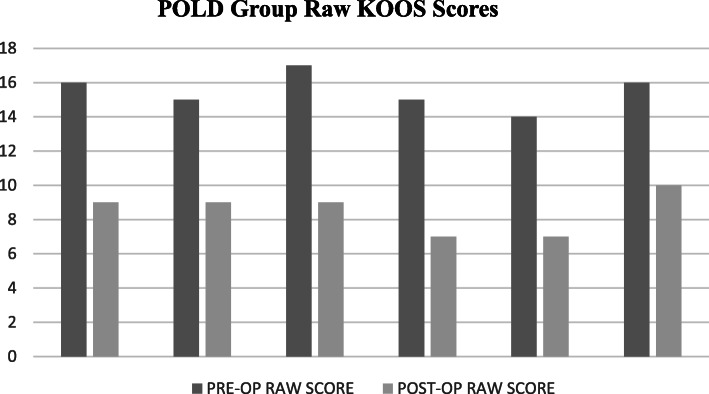
Pre- and postoperative KOOS Jr. of POLD group

The preoperative and postoperative mean KOOS Jr. of PRLD group were 48.73 ± 2.64 and 64.91 ± 2.74, respectively (*p* < 0.001*).* The pre- and postoperative scores of POLD group were 48.83 ± 2.83 and 67.84 ± 4.31 (*p* < 0.001), respectively. The KOOS Jr. of the groups are shown in Figs. 1 and 2. The intergroup variables are summarized in Table 2.

**Table 2:** Intergroup comparison of pre- and postoperative KOOS Jr. score

Variables	PRLD group	POLD group	***p*** Value
Median preoperative RAW KOOS score	15	16	0.637
Median postoperative RAW KOOS score	9	9	0.647
Mean preoperative interval KOOS score	48.73 ± 2.64	48.83 ± 2.83	0.328
Mean postoperative interval KOOS score	64.91 ± 2.74	67.84 ± 4.31	0.329

Intergroup comparisons of pre- and postoperative KOOS Jr. revealed no significant differences (*p* > 0.05). Patients of both groups reported an improvement in physical function and activities of daily living compared with the preoperative status. Wound infection, symptomatic deep vein thrombosis, and other adverse events were not found.

## Discussion

There are many guidelines for TKA and postoperative managements [12]. However, there is a lack of specific guidelines for rehabilitation protocol during the COVID-19 lockdown [3, 5]. The lack of instruction by trained physiotherapists adds more challenges to the patients after discharge [5, 13]. In the first few days of lockdown, there were utter chaos and confusion. The established protocols were side-lined and the patients mainly cared on their own on the dictum of “stay home and stay safe”. Caregivers, the surgeons and therapists alike, provided home-based suggestions based on non-physical assessment *as per* the government guidelines via telephone [10, 14, 15]. Assessing the functional outcomes is important to streamline the further rehabilitation.

There are many evaluation tools to consider. The Oxford knee score, Western Ontario and McMaster Universities Osteoarthritis Index, Patient Reported Outcome Measures, and KOOS Jr. have been used to assess knee osteoarthritis and outcomes of TKA [10]. The Oxford Knee Score is a validated score that is easy to implement due to its comparatively small size. It also assesses hip or spine but with a low specificity. The Western Ontario and McMaster Universities Osteoarthritis Index has drawbacks of cultural disconnection and language barriers in the Hindi speaking population [16, 17]. The Patient Reported Outcome Measures is designed to assess the whole body, in terms of evidence and outcomes, rather than the knee joint alone.

Our assessments included both subjective and objective items. They have easy applicability without compromising the assessment accuracy of functional outcomes. The KOOS Jr. is a knee-specific patient-reported evaluation tool that provides important information from the patient about the impact of knee osteoarthritis and other knee disorders and the treatment efficacy [18]. It is relatively shorter with a higher responsiveness and consistency, especially its scoring methodology is easy to use [10]. The KOOS Jr. is limited to subjects with relatively lower activity demand (with going up and down stairs being the most rigorous activities) and are more difficult to use in older, less-active adults [9]. We routinely used the KOOS Jr. to evaluate the outcomes of TKA. As a validated measurement, the KOOS Jr. scoring does not require any outdoor activity and can be derived from a longer survey [11]. The scoring involves frequently touching the patients and using devices and machines. However, our study found the trained home caregivers can also effectively and reliably score function of the knee through the telephone or video conversation, especially during the period of COVID-19 lockdown.

The role of a physical therapist or a home caregiver is crucial in the medium- to long-term response to assist the patient to regain optimal function, and to help them return to the previous level of activities. We suggest that home caregivers should be trained and educated with rehabilitation devices based on the protocol. We suggest documents and communication links should be provided for future learning programs.

Patient satisfaction scoring is a self-objective assessment of TKA, which is about 80% based on the surgeon’s objective assessment [18, 19]. The Performance-Based Outcome Measures may not truly reflect the knee function, because it is influenced by socioeconomic, cultural, and psychological factors [18, 20]. Despite the limitations of performance-based assessments, it can provide more useful information than the existing patient- or physician-assessed questionnaires [18]. Social isolation and loneliness of the elderly patients affected the psychological aspect and, as a result, may delay the recovery. During lockdown, non-availability of domestic help and poor communications limit the patient’s telecommunication interactions [13]. After released from the hospital, the patients should be assisted by the physical therapists or home caregivers to keep morale at a higher level during the challenging period.

Rehabilitation should be implemented immediately after surgery. The patients were tracked to prevent the loss of follow-up [5]. Video conferencing may lack physical examination and is very likely fraught with misunderstandings. Coordination among the surgeon, therapists, and home caregivers is critical to ensuring the continued rehabilitation during lockdown [5]. We help the patients develop realistic expectations about the impact of TKA [8].

This study has limitations. It is a retrospective study with small sample size and randomization was impossible. A longer follow-up period is required to better ascertain the long-term viability of rehabilitation [20]. Future studies should be blinded and randomized. Surgeon, therapist, and home caregiver experience and ability may influence results of the outcome assessments.

## Conclusion

Although the lockdown due to COVID-19 pandemic affected the routine postoperative rehabilitation after TKA, the coordination among the surgeon, therapists, and home caregivers can provide continued assistance in rehabilitation. The guidelines for practitioners and physiotherapists benefit functional recovery of the patients after TKA.

## Data Availability

The datasets used and/or analysed during the current study are available from the corresponding author on request.

## Abbreviations

TKA: Total knee arthroplasty
OA: Osteoarthritis
PRLD: Pre-lockdown
POLD: Post Lockdown
KOOS JR: Knee injury and osteoarthritis outcome score, Junior
SPSS: Statistical package for social sciences
ANOVA: Analysis of variance

## References

[1] Ranawat CS, Ranawat AS, Mehta A. Total knee arthroplasty rehabilitation protocol: What makes the difference? J Arthroplasty. 2003;18(3):27–30. 10.1054/arth.2003.50080. 12730924 10.1054/arth.2003.50080

[2] Moffet H, Collet JP, Shapiro SH, Paradis G, Marquis F, Roy L. Effectiveness of Intensive Rehabilitation on Functional Ability and Quality of Life After First Total Knee Arthroplasty: A Single-Blind Randomized Controlled Trial. Arch Phys Med Rehabil. 2004;85:546–56. 10.1016/j.apmr.2003.08.080. 15083429 10.1016/j.apmr.2003.08.080

[3] Neradi D, Hooda A, Shetty A, Kumar D, Salaria AK, Goni V. Management of Orthopaedic Patients during COVID-19 pandemic in India: a guide. Indian J Orthop. 2020;54:402–7. 10.1007/s43465-020-00122-6. 32341601 10.1007/s43465-020-00122-6 PMC7184166

[4] BagariaV SD. Orthopaedics in Times of COVID 19. Indian J Orthop. 2020;54:400–1. 10.1007/s43465-020-00123-5. 32341600 10.1007/s43465-020-00123-5 PMC7184164

[5] Editorial: A Message from the Nepal Physiotherapy Association: The Role of Physical Therapists in the Medical Response Team Following a Natural Disaster: Our Experience in Nepal. J Orthop Sports Phys Ther. 2015;45(9):644–646. doi:10.2519/jospt.2015.0108. 10.2519/jospt.2015.0108 26323564

[6] Sherman DW. A review of the complex role of family caregivers as health team members and second -order patients. Healthcare (Basel). 2019;7(2):63. 10.3390/healthcare7020063. 31022837 10.3390/healthcare7020063 PMC6627519

[7] Saklad M. Grading of patients for surgical procedure. Anesthesiology. 1941;2:281–4. 10.1097/00000542-194105000-00004.

[8] Rodriguez-Merchan EC. Knee instruments and rating scales designed to measure outcomes. J Orthopaed Traumatol. 2012;13:1–6. 10.1007/s10195-011-0177-4. 10.1007/s10195-011-0177-4 PMC3284660 22274914

[9] Lyman S, Lee Y-Y, Franklin PD, Li W, Cross MB, Padgett DE. Validation of the KOOS, JR: a short-form knee Arthroplasty outcomes survey. Clin Orthop Relat Res. 2016;474:1461–71. 10.1007/s11999-016-4719-1. 26926773 10.1007/s11999-016-4719-1 PMC4868168

[10] Guide on Knee PRO Measures by codetechnology. https://www.codetechnology.com. Accessed 30 May 2020.

[11] Board of Governors. In supersession of the Medical Council of India: Telemedicine practice guidelines enabling registered medical practitioners to provide healthcare using telemedicine. https ://www.mohfw.gov.in/pdf/Telemedicine.pdf. Accessed 30 May 2020.

[12] Chhabra HS, Bagaraia V, Keny S, Kalidindi KKV, Mallepally A, Dhillon MS, et al. COVID-19: current knowledge and best practices for orthopaedic surgeons. Indian J Orthop. 10.1007/s43465-020-00135-1. 10.1007/s43465-020-00135-1 PMC7232909 32425237

[13] Smith T. “On their own”: social isolation, loneliness and chronic musculoskeletal pain in older adults. Qual Ageing Older Adults. 2017;18(2). doi:10.1108/QAOA-03-2017-0010.

[14] Small SR, Bullock GS, Khalid S, Barker K, Trivella M, Price AJ. Current clinical utilisation of wearable motion sensors for the assessment of outcome following knee arthroplasty: a scoping review. BMJ open. 2019;9(12). doi:10.1136/ bmjopen-2019-033832. 10.1136/bmjopen-2019-033832 PMC6936993 31888943

[15] Abel K, APN, Baldwin K, Chuo J, Wells LM, Ganley TJ, Kim A, Giordano T. Can Telemedicine Replace the First Post Op Visit for Knee Arthroscopy in Adolescents? JBJS Orthopaedics Phys Assistants. 2017;5(4):e26. doi:10.2106/jbjs.jopa.17.00014.

[16] Gogtay NJ, Thatte UM, DasGupta B, Deshpande S. Use of the WOMAC questionnaire in Mumbai and the challenges of translation and cross-cultural adaptation. Indian J Med Ethics. 2016;10:33–5. 10.20529/IJME.2013.007. 10.20529/IJME.2013.007 23439195

[17] Sharma J, Khan SA, Rustgi S. Translation and validation of the reduced Western Ontario and McMaster universities osteoarthritis index (WOMAC) in Hindi speaking Indian patient with osteoarthritis of knee. Indian J Physiother Occup Ther Int J. 2010;4:37–41.

[18] Konan S, Hossain F, Patel S, Haddad FS. Measuring function after hip and knee Surgery-the evidence to support performance-based functional outcome tasks. Bone Joint J. 2014;96-b:1431–5. 10.1302/0301-620X.96B11.33773. 25371452 10.1302/0301-620X.96B11.33773

[19] Scott CEH, Howie CR, MacDonald D, Biant LC. Predicting dissatisfaction following total knee replacement- a prospective study of 1217 patients. J Bone Joint Surg. 2010;92(9):1253–8. 10.1302/0301-620X.92B9.24394. 10.1302/0301-620X.92B9.24394 20798443

[20] Luna IE, Kehlet H, Peterson B, Wede HR, Hoevsgaard SJ, Aasvang EK. Early patient-reported outcomes *versus* objective function after total hip and knee arthroplasty- a prospective cohort study. Bone Joint J. 2017;99-B:1167–75. 10.1302/0301-620X.99B9.BJJ-2016-1343.R1. 28860396 10.1302/0301-620X.99B9.BJJ-2016-1343.R1

